# Effect of Rotational Speed on the Resistance of NiTi Alloy Endodontic Rotary Files to Cyclic Fatigue—An In Vitro Study

**DOI:** 10.3390/jcm11113143

**Published:** 2022-05-31

**Authors:** Vicente Faus-Matoses, Vicente Faus-Llácer, Celia Ruiz-Sánchez, Sharon Jaramillo-Vásconez, Ignacio Faus-Matoses, Benjamín Martín-Biedma, Álvaro Zubizarreta-Macho

**Affiliations:** 1 Department of Stomatology, Faculty of Medicine and Dentistry, University of Valencia, 46010 Valencia, Spain; vicente.faus@uv.es (V.F.-M.); fausvj@uv.es (V.F.-L.); ceruizsan@gmail.com (C.R.-S.); sjavas@alumni.uv.es (S.J.-V.); 2 Department of Surgery and Medical-Surgical Specialties, School of Medicine and Dentistry, Universidad de Santiago de Compostela, 15705 Santiago de Compostela, Spain; benjamin.martin@usc.es; 3 Department of Implant Surgery, Faculty of Health Sciences, Alfonso X El Sabio University, 28691 Madrid, Spain; amacho@uax.es; 4 Department of Surgery, Faculty of Medicine and Dentistry, University of Salamanca, 37008 Salamanca, Spain

**Keywords:** continuous rotation, cyclic fatigue, endodontics, endodontic rotary file, reciprocating, speed, resistance

## Abstract

The present study aims to evaluate and contrast the function of the rotational speed of NiTi alloy endodontic rotary files on how resistant they are to dynamic cyclic fatigue. Methods: A total of 150 NiTi alloy endodontic rotary files with similar geometrical design and metallurgical properties were randomly divided into study groups: Group A: 200 rpm (*n* = 30); Group B: 350 rpm (*n* = 30); Group C: 500 rpm (*n* = 30); Group D: reciprocating movement at 350 rpm with 120° counterclockwise and 30° clockwise motion (350 rpm+) (*n* = 30); and Group E: reciprocating movement at 400 rpm with 120° counterclockwise and 30° clockwise motion (400 rpm+) (*n* = 30). A dynamic device was designed to carry out dynamic cyclic fatigue tests using artificial root canal systems made from stainless steel with an apical diameter of 250 µm, 5 mm radius of curvature, 60° curvature angle, and 6% taper, and 20 mm in length. A Weibull statistical analysis and ANOVA test were used to analyze the results. Results: The ANOVA analysis showed differences in time to failure among all the study groups that were of statistical significance (*p* < 0.001). Conclusions: NiTi alloy endodontic rotary files using reciprocating movement at 350 rpm with 120° counterclockwise and 30° clockwise motion exhibit greater resistance to dynamic cyclic fatigue than files used with a reciprocating movement at 400 rpm with 120° counterclockwise and 30° clockwise motion, continuous rotational speed at 200 rpm, continuous rotational speed at 350 rpm, or continuous rotational speed at 500 rpm; it is therefore advisable to use reciprocating movements at a low speed.

## 1. Introduction

Chemical disinfection and mechanical instrumentation of the root canal system are crucial in the prevention of apical periodontitis that arises due to treatment, or to cure it if already established [1]. However, the failure of nickel–titanium (NiTi) alloy endodontic rotatory files remains a major dilemma for endodontists during root canal treatment, despite the NiTi alloy undergoing continuous chemical and mechanical enhancements by manufacturers so as to help prevent complications during endodontic therapy [2]. The fracture of NiTi alloy endodontic rotary files can be caused by torsional fatigue, cyclic fatigue, or some combination thereof [3]. Torsional failure happens when the end of a NiTi alloy endodontic rotary file has become trapped on one of the root canal walls while the instrument is still rotating, causing the file to fracture once the elasticity of the material has been exceeded [4,5]. Flexural bending fatigue is caused by the repeated application of compression and traction cycles that the NiTi alloy endodontic rotary file experiences at the site of maximum curvature of the root canal; these stresses subsequently lead to plastic deformation, which can result in unexpected file fracture [3,6].

Several studies have reported that a fractured fragment of the NiTi alloy endodontic rotary file may block the curved canal, negatively affecting the treatment outcome, as disinfecting agents can no longer reach the infected root canal areas [1,7,8]. Additionally, root canal systems that have not been properly disinfected may have a lower likelihood of healing in teeth with periapical lesions [9].

Several additional factors have been linked to the fracture of NiTi alloy endodontic rotary files, including instruments with a cross-section design [10], taper and apical diameter [11], flute length, pitch, and helix angle [12]. In addition, the dynamics of the instrument, such as torque [13] and canal geometry [8], as well as the manufacturing process, whether electropolishing, heat treatment, or ion implantation [14], can influence the risk of fracture.

It remains unclear whether or not rotational speed affects the resistance to cyclic fatigue of NiTi alloy endodontic rotary files. Yared et al. and Martín et al. have found that rotational speed does indeed influence the prevalence of fracture in NiTi alloy endodontic rotary files [15,16]. However, Pruett et al. showed that rotational speed had no significant impact on the risk of fracture of NiTi alloy endodontic rotary files [8]. Additionally, some studies have reported that reciprocating motion may overextend the cyclic fatigue life of NiTi alloy endodontic files in comparison to continuous motion [17,18].

The present study aims to evaluate and assess the effect of the rotational speed of NiTi alloy endodontic rotary files on their resistance to dynamic cyclic fatigue, with a null hypothesis (H_0_) postulating that rotational speed has no effect on how resistant NiTi alloy endodontic rotary files are to dynamic cyclic fatigue.

## 2. Materials and Methods

### 2.1. Study Design

One hundred and fifty (150) sterile, brand new endodontic rotary files with a parallelogram cross-section design, 6% taper, and 250 µm apical diameter (Ref.: IRE 02506, D, Endogal, Galician Endodontics Company, Lugo, Spain) were randomly distributed among different study groups: Group A: continuous rotational speed at 200 rpm (200 rpm) (*n* = 30); Group B: continuous rotational speed at 350 rpm (350 rpm) (*n* = 30); Group C: continuous rotational speed at 500 rpm (500 rpm) (*n* = 30); Group D: reciprocating movement at 350 rpm with 120° counterclockwise and 30° clockwise motion (350 rpm+) (*n* = 30); and Group E: reciprocating movement at 400 rpm with 120° counterclockwise and 30° clockwise motion (400 rpm+) (*n* = 30). The final total of experimental units included was 150, with these being assigned to one of the five study groups in keeping with the proportions determined by the researchers. The power was set at 80% and testing the null hypothesis H_0_ resulted in an effect size of 0.606. A single-factor ANOVA test for independent samples was used to make equal the mean values of the five groups, and the significance level was set at 5%. A microscope (OPMI pico, Zeiss, Oberkochen, Germany) was used to examine all NiTi alloy endodontic rotary files (Ref.: IRE 02506, D, Endogal, Galician Endodontics Company, Lugo, Spain) prior to use, with no files discarded. Between January and July 2022, this controlled experiment was conducted at the Department of Stomatology of the Faculty of Medicine and Dentistry at the University of Valencia (Valencia, Spain).

### 2.2. Analysis with Scanning Electron Microscopy

A scanning electron microscope (SEM) (HITACHI S-4800, Fukuoka, Japan) was used at ×30 and ×600 for the initial inspection of the NiTi alloy endodontic rotary files (Ref.: IRE 02506, D, Endogal, Galician Endodontics Company, Lugo, Spain). This analysis was conducted at the Central Support Service for Experimental Research of the University of Valencia in Burjassot, Spain. The analysis was carried out with the following exposure parameters: 20 kV acceleration voltage; magnification from 100× to 6500×; and resolution ranging from −1.0 nm at 15 kV to 2.0 nm at 1 kV. Researchers did this to evaluate the surface characteristics and ensure there were no manufacturing surface defects.

### 2.3. Analysis with Energy-Dispersive X-ray Spectroscopy

In addition, energy-dispersive X-ray spectroscopy (EDX) was also used to analyze all the NiTi alloy endodontic rotary files (Ref.: IRE 02506, D, Endogal, Galician Endodontics Company, Lugo, Spain). This was conducted at the Central Support Service for Experimental Research at the University of Valencia in Burjassot, Spain. This inspection used these exposure parameters: 20 kV acceleration voltage; magnification from 100× to 6500×; and resolution ranging from −1.0 nm at 15 kV to 2.0 nm at 1 kV. These parameters were used to assess the elemental makeup of the chemicals in the files used to test their resistance to static fatigue. The researchers also evaluated the atomic weight percent, taking measurements from three different sections (apical third, medium third, and coronal third of the NiTi alloy endodontic files).

### 2.4. Experimental Model Simulating Dynamic Cyclic Fatigue

**Figure 1:**
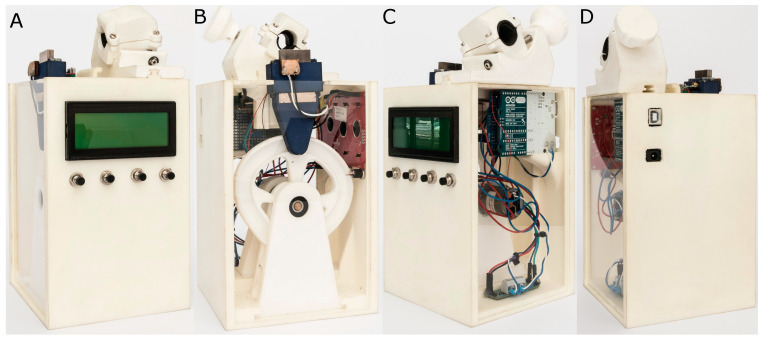
(**A**) Front, (**B**) back, (**C**) right, and (**D**) left sides of the dynamic cyclic fatigue device.

The researchers conducted tests of resistance to dynamic cyclic fatigue at room temperature (20 °C) to evaluate the mechanical behavior of the instruments, according to Martins et al. [19], using the aforementioned customized device (Utility Model Patent No. ES1219520) [20]. CAD/CAE 2D/3D software (Midas FX+^®^, Brunleys, Milton Keynes, UK) was used to design the structure of the device, which was subsequently created with 3D-printing software (ProJet^®^ 6000 3D Systems©, Rock Hill, SC, USA) (Figure 1).

The customized artificial root canals were performed using Schneider’s measuring technique, with a curvature of 60° [21] and a 5 mm curvature radius. The inverse engineering software used for this purpose was CAD/CAE 2D/3D. Molybdenum wire-cut technology (Cocchiola S.A., Buenos Aires, Argentina) was used with electrical discharge machining (EDM) to create the artificial root canal from stainless steel. Researchers also ensured that the NiTi files were flush with the walls of the artificial root canal. This newly created artificial canal was then positioned on its support, and a light-dependent resistor (LDR) sensor (Ref.: C000025, Arduino LLC^®^, Ivrea, Italy) placed at the apex of the canal was used to identify any failures in the endodontic rotary instruments (Ref.: IRE 02506, D, Endogal, Galician Endodontics Company, Lugo, Spain). This device works by measuring the light source continuously generated by a very strong white LED (20,000 mcd) (Ref.: 12.675/5/b/c/20k, Batuled, Coslada, Spain). The LED was positioned opposite the artificial root canal. An LED LDR sensor (Ref.: C000025, Arduino LLC^®^) at 50 ms was used to interpret the LED signals so as to identify the precise time of failure.

A roller bearing system (Ref.: MR104ZZ, FAG, Schaeffler Herzogenaurach, Herzogenaurach, Germany) was used to apply the movement direction and speed indicated by the operator (Ref.: DRV8835, Pololu^®^ Corporation, Las Vegas, NV, USA) and created by the brushed DC gear motor (Ref.: 1589, Pololu^®^ Corporation, Las Vegas, NV, USA) to the artificial support. The support was maneuvered in an exclusively axial motion with the help of a linear guide (Ref.: HGH35C 10249-1 001 MA, HIWIN Technologies Corp. Taichung, Taiwan). A torque-controlled motor and 6:1 reduction handpiece (X-Smart plus, Dentsply Maillefer, Baillagues, Switzerland) were used in conjunction with the NiTi endodontic rotary files.

A frequency of 60 pecks per minute was used for the NiTi endodontic files within the dynamic cyclic fatigue device, following the parameters of a prior study [19]. Researchers also applied a high-flow synthetic oil (Singer All-Purpose Oil; Singer Corp., Barcelona, Spain) to help prevent friction between the NiTi endodontic files and the walls of the artificial root canal; this oil is specifically formulated for the lubrication of mechanical parts.

The files were all used until failure. The researchers recorded and evaluated both the length of time and the number of cycles the files took to fracture.

### 2.5. Statistical Tests

Statistical analysis of all variables was performed using SAS 9.4 (SAS Institute Inc., Cary, NC, USA). The mean value and SD were used to express the descriptive statistics of the quantitative variables. The researchers then used an ANOVA test to perform a comparative analysis of the number of cycles to failure and the time to failure (in seconds). In 2-to-2 comparisons, the Tukey method was used to determine the *p*-values and correct any Type I errors. The researchers also calculated the Weibull modulus and Weibull characteristic strength. Statistical significance was defined as *p* ˂ 0.05.

## 3. Results

**Figure 2:**
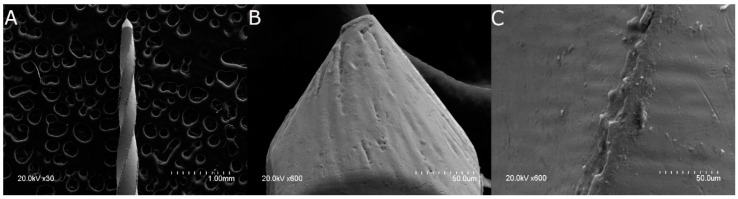
(**A**) SEM images of the full-length NiTi alloy endodontic rotary files (Ref.: IRE 02506, D, Endogal, Galician Endodontics Company, Lugo, Spain) at ×30, (**B**) and specifically of the end of the file at ×600 and (**C**) the surface of the file at ×600.

Scanning electron microscopy (SEM) analysis of the NiTi alloy endodontic rotary files did not detect any structural alterations or accumulated organic matter. Additionally, due to the laser machining process used to make them, the manufacturing lines were parallel to each other and perpendicular to the longitudinal axis of the files. The distance and width between these manufacturing lines were indicators of the precision and intensity of the laser machining manufacturing process. The laser machining process also resulted in tubular porosity that was observed in the files. Additionally, tubular porosity was visible in all of the NiTi alloy endodontic rotary files as a result of the combination of other chemical elements with the Ti alloys (Figure 2).

**Figure 3:**
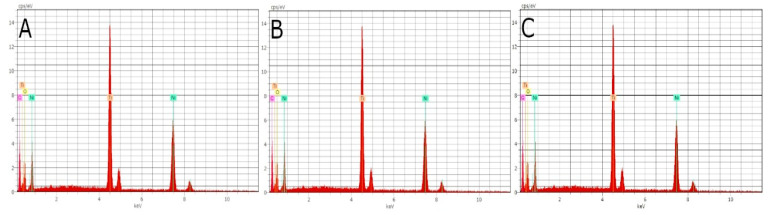
EDX micro-analysis of the NiTi alloy endodontic rotary files at locations (**A**) 1, (**B**) 2, and (**C**) 3.

EDX micro-analysis of the NiTi alloy endodontic rotary files was performed at three different locations at 20 kV, enabling a thorough and precise analysis of the composition of the NiTi alloy endodontic rotary files. Through EDX micro-analysis at 20 kV, the NiTi alloy endodontic rotary files were found to comprise Ti (37.59–34.52 wt.%) and Ni (34.19–38.81 wt.%), although O and C were also observed (Figure 3).

**Figure 4:**
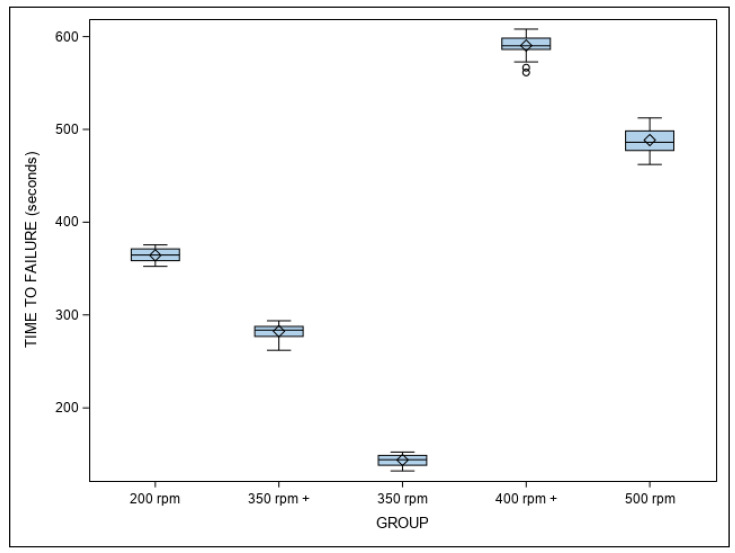
Box plot of time to failure. The median value of the respective study groups is represented by the horizontal line in each box. ◊—Box plot mean value. O—Extrema value.

Table 1 and Figure 4 show the mean and SD values of the time to failure (in seconds) across all study groups.

**Figure 5:**
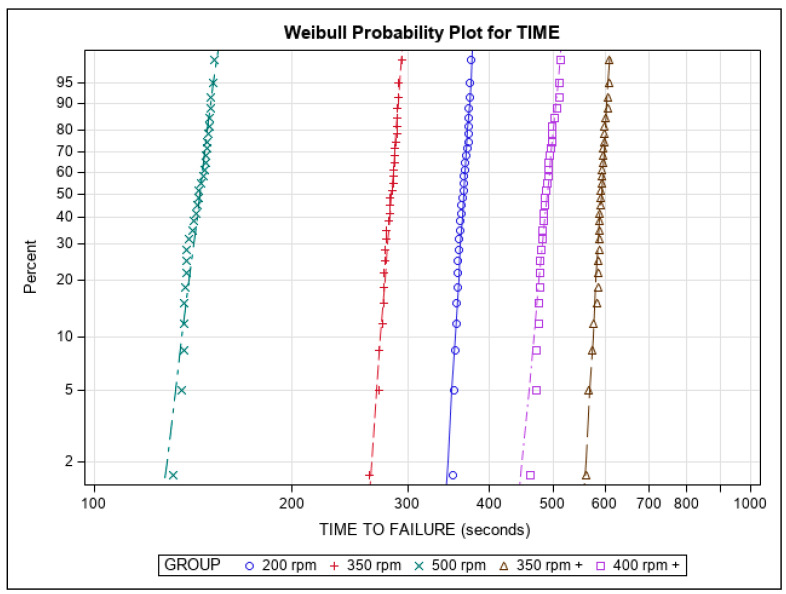
Weibull probability plot displaying time to failure across study groups.

The ANOVA analysis found there were differences of statistical significance among all of the study groups with regard to the time to failure (*p* < 0.001) (Figure 5). The results of the time to failure could be applied to the “number of cycles to failure” since all of the NiTi endodontic files were used at a frequency of 60 pecks per minute within the dynamic cyclic fatigue device.

The Weibull statistics scale distribution parameter (η) identified differences of statistical significance among all of the study groups with regard to the time to failure (*p* < 0.001) (Table 2, Figure 5). The Weibull statistics shape distribution parameter (β) revealed differences great enough to be statistically significant with regard to time to failure between the 200 rpm and 400 rpm+ groups (*p* = 0.0236), the 500 rpm and 350 rpm+ groups (*p* = 0.0003), the 350 rpm+ and 400 rpm+ groups (*p* = 0.0154), the 350 rpm and 500 rpm groups (*p* = 0.0152), and the 200 rpm and 500 rpm groups (*p* = 0.0005). However, there were not enough differences observed in the time to failure between the 350 rpm and 400 rpm+ groups (*p* = 0.2283), the 500 rpm and 400 rpm groups (*p* = 0.1908), the 200 rpm and 350 rpm+ groups (*p* = 0.08925), the 350 rpm and 350 rpm+ groups (*p* = 0.2492), and the 200 rpm and 350 rpm groups (*p* = 0.3123) to be statistically significant (Table 2, Figure 5). In short, the NiTi alloy endodontic rotary systems exhibited very predictable behavior, as it took about the same amount of time for the majority of the endodontic rotary files within each study group to reach the point of failure. The more gradual slope seen when using the NiTi endodontic rotary files at 350 rpm+ would indicate that this behavior is easier to predict than other kinematics. The NiTi alloy endodontic rotary files at 350 rpm+ were shown to be the most resistant to cyclic fatigue, followed by the NiTi alloy endodontic rotary files at 400 rpm+, 200 rpm, 350 rpm, and 500 rpm.

## 4. Discussion

The findings of the present study do not accept the null hypothesis (H_0_), which postulates that rotational speed does not affect the dynamic fatigue resistance of NiTi alloy endodontic rotary files.

The present study used the same NiTi alloy endodontic instruments in rotary and reciprocating kinematic motion since the manufacturer reported that the geometrical design of the NiTi alloy endodontic files allows for its use in both kinematic movements; therefore, manufacturers recommend its use with both continuous and reciprocating rotations. Furthermore, other instrumentation systems can be used with continuous or reciprocating rotation, and it is necessary to have a motor in which the angles can be adjusted. Clear examples can be found in the studies of Yared 2008 [22] and De Deus 2010 [17], where they used instruments that cut clockwise in a reciprocating mode.

Previous studies have analyzed the effects of rotational speed on the number of cycles to fracture of rotary NiTi instruments. Lopes et al. subjected ProTaper Universal instruments F3 and F4 to 300 and 600 rpm; however, the speed values selected were too distant, a cylindrical tube was used as the artificial root canal, and the fracture detection of the NiTi alloy endodontic rotary files was subjective and therefore imprecise. Furthermore, they did not carry out any additional measurement methods [23]. Additionally, some reviews have been conducted with the aim of analyzing the mechanical and metallurgical behavior of endodontic instruments under different testing conditions and methodologies [24,25,26].

The results derived from the present study indicate that the resistance of NiTi alloy endodontic rotary files to cyclic fatigue is inversely proportional to the rotational speed. In addition, reciprocating movements were shown to be more resistant to cyclic fatigue when compared with continuous rotational movements. Moreover, the results derived from the present study present a direct application to the clinical setting, since the reciprocating systems provided higher resistance to cyclic fatigue, followed by the lower values of rotational speed. Therefore, clinicians should choose reciprocating motion systems or reduce the rotational speed of the endodontic torque-controlled motor if the NiTi endodontic rotary or reciprocating file is expected to experience high cyclic fatigue, particularly in root canal systems with a pronounced angle and/or curvature radius.

Specification #28 of the American Dental Association/American National Standards Institute (ADA/ANSI) outlines tests used to measure how flexible stainless steel hand files are, as well as their strength under torsion. These same tests were also adopted under ISO 3630/1, which is meant for instruments with a 0.02 ISO taper. Currently, there are still no specifications or international standards with regard to testing the resistance of endodontic rotary instruments to cyclic fatigue [27]. The ideal model would entail curved canals being instrumented in natural teeth. That being said, each tooth can only be used once with these tests, and instrumentation causes changes to the shape of the root canal, rendering it impossible to establish standardized experimental conditions. Therefore, various methods and devices have been used to analyze the in vitro resistance of NiTi rotary endodontic instruments to cyclic fatigue fractures [28]. Cyclic fatigue is considered a dynamic event itself since the movement of the NiTi alloy endodontic rotary or reciprocating instruments inside the root canal system gives it dynamism. Cyclic fatigue tests have been carried out in a static model under well-controlled experimental conditions; however, the novel pecking movement of the endodontic handpiece of the present cyclic fatigue device provides an additional dynamic movement more representative of the in-and-out motion made by the operator. That being said, studies have shown that the number of cycles to failure is significantly higher in the dynamic model, regardless of the brand or manufacturing processes [29,30,31]. In the static testing model, there is no up-and-down movement applied to the instrument, causing stresses to accumulate at a fixed point. With the dynamic model, however, these stresses are spread out along the full length of the instrument, thereby increasing its cyclic fatigue resistance [23]. Furthermore, researchers have found that the up-and-down motion should not exceed 1, 2, or 3 mm/s in the dynamic testing model so as to simulate clinical conditions [24]. An automatic detection system can be used to identify the precise point of failure of endodontic rotary files [19]. Given this, the present study used an anatomically based artificial root canal design in accordance with Schneider’s method [20], using a 60° curvature angle and radius of 5 mm, and modifying the geometry to adapt to the NiTi endodontic rotary files used in this study [11].

The findings of this study corroborate the findings of Kim et al., who found that the Reciproc R25 and WaveOne Primary heat-treated NiTi alloy endodontic reciprocating files were more resistant to torsion and cyclic fatigue when compared with ProTaper F2 NiTi alloy endodontic rotary files used under continuous rotation [32]. Similarly, De Deus et al. found that the ProTaper F2 NiTi alloy endodontic rotary file also exhibited significantly greater resistance to cyclic fatigue when employed using reciprocating movement rather than continuous rotational motion [17]. Furthermore, several other studies have emphasized the increase in the lifespan of NiTi alloy endodontic rotary files when using reciprocating movement as opposed to continuous rotational motion [33,34]. That being said, there are several studies that have analyzed the impact of rotational speed on how resistant NiTi alloy endodontic rotary files are to cyclic fatigue, although the findings remain controversial. Lopes et al. found that the ProFile NiTi alloy endodontic rotary instrument exhibited greater susceptibility to accidental fracture at higher rotational speeds, and they found that the total number of cycles to failure was about 30% lower in ProTaper instruments when the rotational speed was increased from 300 to 600 rpm [23]. On the other hand, Martin et al. reported that unexpected fracture of NiTi alloy endodontic rotary instruments was correlated with the rotational speed, as the ProTaper NiTi alloy endodontic rotary instrument was more susceptible to fracture at 350 rpm than at 250 or 150 rpm [16]. However, Gao et al. reported no statistically significant differences (*p* > 0.05) between files that had similar NiTi alloys and apical diameters when used at different rotational speeds [35]. The discrepancies in these findings may be due to differing study designs, NiTi alloys, or geometrical designs of the instruments under study. Additionally, not only the asymmetric oscillatory counterclockwise motion (reciprocation motion) but also the asymmetric oscillatory clockwise motion can be used with any rotary instrument. Martins et al. evaluated the cyclic fatigue resistance of three replicate rotary instruments compared with their original brand systems using continuous rotation and optimum torque reverse kinematics. They reported that reciprocating files showed greater resistance to cyclic fatigue than continuous rotation files, and the replicas showed higher cyclic fatigue resistance than the original brand instruments and higher transition temperatures to the austenitic phase [36].

The results found by Ray et al. were corroborated by those obtained in the present study using an analysis of dynamic cyclic fatigue when employing a standardized axial movement, increasing the durability of NiTi alloy endodontic rotary instruments subjected to cyclic fatigue in comparison with the results observed in static cyclic fatigue devices [37]. Most studies comparing dynamic and static cyclic fatigue appliances have concluded endodontic rotary instruments exhibited a time to fracture roughly 20–40% longer when undergoing dynamic cyclic fatigue than the time to fracture found in studies of static cyclic fatigue, with this also being more similar to the clinical setting [38,39,40].

The cyclic fatigue testing was performed in a room temperature setting, according to the results by La Rosa et al., who showed that studies at body temperature impaired the cyclic fatigue resistance of most files [41]. In addition, Plotino et al. reported that the surrounding temperature affected the NiTi crystalline phase transformation, significantly decreasing the cyclic fatigue resistance at body temperature [42].

Regrettably, the limitations of this study precluded analyzing any additional kinematic movements, under both reciprocating and continuous rotation movements. Future studies ought to include more NiTi alloys, apical diameters, pitch, helix angles, manufacturing processes, and tapers. Furthermore, due to difficulties with the standardization of samples, the present study was not conducted in a clinical setting. However, the present study provided multimethod research, including SEM, EDX, and an accurate dynamic cyclic fatigue device, increasing the knowledge of the mechanical behavior of NiTi endodontic rotary files under different kinematic conditions.

## 5. Conclusions

NiTi alloy endodontic rotary files used with a reciprocating movement at 350 rpm with 120° counterclockwise and 30° clockwise motion were more resistant to dynamic cyclic fatigue than those used with a reciprocating movement at 400 rpm with 120° counterclockwise and 30° clockwise motion, continuous rotational speed at 200 rpm, continuous rotational speed at 350 rpm, and continuous rotational speed at 500 rpm. It is therefore advisable to use reciprocating movements at a low speed.

## Figures and Tables

**Table 1:** Descriptive analysis of time to failure (seconds).

Study Group	*n*	Mean	SD	Minimum	Maximum
200 rpm	30	364.30 ^a^	6.71	352.38	375.49
350 rpm	30	282.42 ^b^	7.19	261.90	293.71
500 rpm	30	143.84 ^c^	5.70	132.08	152.39
350 rpm+	30	590.38 ^d^	11.19	561.37	608.08
400 rpm+	30	488.44 ^e^	12.93	462.19	512.33

^a,b,c,d,e^ Statistically significant differences among groups (*p* < 0.05).

**Table 2:** Weibull statistics for the time to failure across the study groups.

Study Group	Weibull Shape (β)				Weibull Scale (η)			
	Estimate	St Error	Lower	Upper	Estimate	St Error	Lower	Upper
200 rpm	61.9124	8.8223	46.8258	81.8598	367.5283	1.1471	365.2868	369.7836
350 rpm	50.3905	7.3394	37.8766	67.0388	285.6319	1.0898	283.5039	287.7759
500 rpm	30.5162	4.4688	22.9024	40.6611	146.4785	0.9251	144.6765	40.6611
350 rpm+	63.6086	8.9083	48.3399	83.7000	595.4815	1.8047	591.9549	599.0291
400 rpm+	39.6357	5.3913	30.3603	51.7449	494.7559	2.4189	490.0376	499.5197

## Data Availability

Information available upon request, subject to relevant restrictions (such as privacy or ethical).
